# Psychosocial Nursing Diagnoses of Individuals With Myalgic Encephalomyelitis‐Chronic Fatigue Syndrome: A Descriptive Study

**DOI:** 10.1002/nop2.70212

**Published:** 2025-04-30

**Authors:** Cristina Oter‐Quintana, Almudena Alameda‐Cuesta, Pedro Ruymán Brito‐Brito, Ana Isabel Parro‐Moreno, María Teresa Alcolea‐Cosín, Teresa González‐Gil, Valentín Hernández‐Barrera, Jesús Esteban‐Hernández

**Affiliations:** ^1^ Doctorate in Health Sciences Faculty of Health Sciences Rey Juan Carlos University Alcorcón Spain; ^2^ Member of the Nursing and Health Care Research Group IDIPHISA Majadahonda Spain; ^3^ Nursing Department, Faculty of Medicine Autonomous University of Madrid Madrid Spain; ^4^ Nursing and Oral Medicine Department, Faculty of Health Sciences Rey Juan Carlos University Alcorcón Spain; ^5^ Nursing Department University of La Laguna Tenerife Spain; ^6^ Medical Specialties and Public Health Department, Faculty of Health Sciences Rey Juan Carlos University Alcorcón Spain

**Keywords:** chronic, cluster analysis, fatigue syndrome, nursing, nursing diagnosis

## Abstract

**Aim:**

To describe the prevalence of psychosocial nursing diagnostic labels and their relationship with sociodemographic characteristics in adults with myalgic encephalomyelitis‐chronic fatigue syndrome (ME/CFS).

**Design:**

This is a cross‐sectional descriptive study.

**Methods:**

Population: Adults with ME/CFS. Inclusion criteria: Being 18 years of age or older, having a medical diagnosis of ME/CFS and being an active member of a patient association. Data collection took place between May and July 2022 using an online and paper‐based ad hoc form that included sociodemographic and clinical data. Psychosocial diagnostic labels were obtained using the Questionnaire for Psychosocial Nursing Diagnosis (QPSND). In addition to a descriptive analysis, the relationships between the diagnostic labels obtained were explored through a multiple correspondence analysis, which was supplemented by a hierarchical cluster analysis of the results of the latter.

**Results:**

Forty‐eight participants completed the form. Their mean age was 52.5 years (SD = 6.81), 95.83% were female, 70.83% had a university education, and 35.42% were actively working. Sixty‐six percent had some degree of officially recognised disability, and 16.67% had an officially recognised degree of dependency. The most prevalent diagnostic labels were Powerlessness (79.17%), Ineffective Coping (62.5%), and Fear (62.5%). The multiple correspondence analysis and subsequent cluster analysis identified profiles of individuals with ME/CFS: one profile (cluster 3) had greater psychosocial involvement based on the diagnostic labels assigned, as well as a lower educational level and higher symptom intensity. The other two profiles appear to bring together mainly employed or retired individuals with lower severity and frequency of symptoms, and who are at risk of developing psychosocial human responses.

**Conclusions:**

Participants have a high prevalence of psychosocial diagnostic labels, suggestive of the psychosocial distress concomitant with ME/CFS. Nursing diagnoses allow subgroups of affected individuals to be differentiated and aligned based on differences in sociodemographic and clinical characteristics.

**Implications for the Profession and/or Patient Care:**

We believe that this is a pioneering study in the identification of psychosocial nursing diagnostic labels of individuals with ME/CFS. Having profiles of people with ME/CFS associated with psychosocial nursing diagnoses facilitates their identification in healthcare practice and makes it possible to anticipate recommended interventions.

**Impact:**

- What problem did the study address?
  ○ This study aims to ascertain the prevalence of psychosocial nursing diagnostic labels in individuals with ME/CFS. It also aims to identify more sociodemographic and clinical characteristics associated with these psychosocial problems.
- What were the main findings?
  ○ Individuals with ME/CFS had a high prevalence of psychosocial nursing diagnostic labels. Three subgroups of participants with ME/CFS were identified based on their diagnostic labels. Characteristics such as lower educational level, higher symptom intensity, and a diagnosis of fibromyalgia and Sjögren's syndrome, in addition to ME/CFS, were associated with the subgroup that had the most adverse psychosocial diagnostic profile. The other two subgroups appear to bring together mainly employed or retired individuals with lower severity and frequency of symptoms and who are at risk of developing certain psychosocial human responses.
- Where and on whom will the research have an impact?
  ○ This study may have an impact on both nursing management and clinical practice by informing the design of care plans for patients with ME/CFS.

**Reporting Method:**

STROBE.

**Patient or Public Contribution:**

Contributions from individuals with ME/CFS were taken into consideration for the study design, especially regarding the sampling and data collection procedures. The results of the study were presented publicly at research conferences attended by health professionals and members of associations of people living with ME/CFS.

## 1 Introduction

Myalgic encephalomyelitis/chronic fatigue syndrome (ME/CFS) is a complex, chronic and multisystemic disease (Bateman et al. 2021) that negatively affects the quality of life of those who suffer from it and those closest to them. Its actual prevalence is unclear, with considerable differences in the figures reported by studies depending on the study population, clinical case definition and diagnostic method employed. Nonetheless, a meta‐analysis by Lim et al. (2020) establishes a prevalence of 0.68% [95% CI = 0.48–0.97], albeit with substantial heterogeneity (*I* ^2^ = 99.4%). ME/CFS is more common in females than in males and can occur in childhood and adolescence, as well as in adulthood (Bateman et al. 2021).

The aetiology of ME/CFS is unknown, and some hypotheses suggest that certain immunological, neurological, intestinal and mitochondrial abnormalities may play a role in its emergence and permanence (Muller et al. 2020). Diagnosis is currently based on detailed clinical records, physical examinations and supplementary tests, with the clinical case definition from the International Consensus Criteria being one of the most widely used (Carruthers et al. 2011). The cardinal symptom is post‐exertional neuroimmune exhaustion (PENE), characterised by a worsening of symptoms following physical and/or mental exertion, which may be minimal and which, before the onset of the disease, was well tolerated. The onset of symptoms may be delayed after the initial trigger, and recovery can take hours, days or even weeks. Individuals experience a significant reduction in their previous activity levels, along with a low threshold for physical and/or mental fatigability. Furthermore, neurological, immune, gastrointestinal and genitourinary impairments, as well as disruptions in energy metabolism and transportation, are commonly observed (Carruthers et al. 2011). ME/CFS often occurs alongside other comorbid conditions, such as fibromyalgia, myofascial pain syndrome, and sicca syndrome, among others, with more than 80% of individuals affected in some cohorts (Castro‐Marrero et al. 2017).

The disease follows an unpredictable course, characterised by symptom fluctuations, sometimes within the same day, and periods of relapse that significantly reduce functional capacity. The degree of functional impairment can vary. In its mildest form, individuals may be able to carry out self‐care activities and participate in light household tasks. They may be able to continue to work or study at the expense of other activities. In its most severe form, individuals require assistance with basic activities of daily living and may remain bedridden at home (Bateman et al. 2021; National Institute for Health and Care Excellence 2021). There is currently no cure for the disease, but the keys to a therapeutic approach are as follows: validating the affected person's experience; assessing their care needs; providing them with the necessary support; treating symptoms and associated comorbidities; and teaching them techniques aimed at preserving and managing their energy to minimise the frequency, duration and severity of the post‐exertional malaise (or post‐exertional neuroimmune exhaustion) characterising this pathology (Bateman et al. 2021).

## 2 Background

The absence of a clear cause of disease symptoms and the existence of different clinical case definitions, together with the co‐occurrence of ME/CFS with other clinical conditions, have led to much debate among health professionals who question the very existence of the disease. As a result, ME/CFS has been considered a ‘contested illness’: a term used, among others, by Swoboda (2006) to refer to a group of illnesses whose very existence is controversial and disputed by public authorities, healthcare professionals and society in general. Although affected individuals experience persistent, debilitating and real symptoms, scepticism and physicians' lack of knowledge about the disease hinders diagnosis, which can take years after the onset of symptoms (Bayliss et al. 2014). It is common to psychologise the symptoms of ME/CFS, attributing them to problems such as depression, stress or somatisation, in which the physical symptoms are an expression of social or emotional discomfort (Geraghty and Blease 2019). Failure to validate the individual's experience of their illness undermines the therapeutic relationship, resulting in an atmosphere of tension and mistrust that negates the sick person as a knowledgeable individual, leading to feelings of frustration, disappointment, anger, fear and loneliness (Bayliss et al. 2014; Alameda Cuesta et al. 2021). Moreover, the contested status of ME/CFS hinders access to social benefits, leaving those affected in a situation of extreme vulnerability and dependency (Alameda Cuesta et al. 2021; de Carvalho Leite et al. 2011).

The loss of functional abilities has an impact on the psychological wellbeing of patients, who experience a loss of self‐worth and a sense of guilt as they feel they are a burden to those closest to them (Williams et al. 2019). The fluctuating nature of the symptoms, together with the lack of legitimisation by the health system, also generates mistrust among relatives, friends, and co‐workers (Dickson et al. 2007). All this contributes to feelings of hopelessness (Devendorf et al. 2020) and distress in those affected, straining (or breaking down) previous social relationships and making it difficult for them to seek help (Williams et al. 2019).

Bury (1982) points out that chronic illness is a disruptive experience in an individual's life that impacts both their physical self and their identity. In this case, ME/CFS involves processes of subjective transformation, which are negatively affected by the contested nature of the disease (Alameda Cuesta et al. 2021).

The areas of interest to the nursing discipline are human responses/experiences of illness (Neuman 2010). As such, by labelling the experiences associated with an illness, nursing diagnoses validate the patient's knowledge of their condition, validation that has therapeutic value for those living with ME/CFS (Bateman et al. 2021; Picariello et al. 2017). In its ninth edition (Herdman 2012), the NANDA‐I nursing diagnoses classification, within the framework of the NNN taxonomy for nursing practice, incorporated the psychosocial domain alongside three others: functional, physiological, and environmental. This domain includes nursing diagnoses, outcomes, and interventions designed to promote optimal mental and emotional wellbeing while supporting effective social functioning. However, few studies have investigated the prevalence of psychosocial nursing diagnoses in specific population groups (Brito and Aguirre 2014; Sanz Sánchez 2017). This research gap is particularly concerning given the potential distress it can cause to affected individuals and the challenges it poses to the development of systematic, targeted care.

The identification of nursing diagnoses in people living with ME/CFS remains underexplored. The few studies that have made progress in diagnostic formulation take the clinical expertise of practitioners into consideration but fail to provide empirical evidence to support such formulations (Royes et al. 2010). Given that the experience of illness of individuals with ME/CFS transcends the symptoms derived from their disease, this study aims to make progress in the identification of psychosocial nursing diagnoses in individuals living with ME/CFS.

## 3 The Study

### 3.1 Aims

This study aims to describe the prevalence of psychosocial diagnostic labels and the relationship pattern between these labels and other sociodemographic characteristics in adults with ME/CFS.

## 4 Methods/Methodology

### 4.1 Design

This is a descriptive cross‐sectional study.

### 4.2 Valid and Reliable Instrument

A bespoke web‐based form was designed for data collection. In addition to sociodemographic information, previously validated questionnaires were included to gather clinical data and assign psychosocial diagnostic labels. Specifically, symptoms experienced in the previous 6 months were collected using a Spanish version of the DePaul Symptom Questionnaire–Short Form (DSQ‐SF) (Jason and Coffin 2022). This 14‐item questionnaire collects highly prevalent symptoms among people with ME/CFS by measuring the frequency and severity of each symptom on a 5‐point Likert scale, ranging from 0—none of the time (frequency)/symptom not present (severity)—to 4—all of the time (frequency)/very severe (severity). Due to its brevity, the DSQ‐SF is an interesting tool to explore symptoms in clinical research, among others (Sunnquist et al. 2019).

The Questionnaire for Psychosocial Nursing Diagnosis (QPSND) was used in the identification of potential psychosocial nursing diagnostic labels (Brito and Aguirre 2014). This instrument assists nurses in identifying up to 28 diagnostic labels in the psychosocial sphere. It consists of 61 items with dichotomous (yes/no) responses and Likert scales (always, often, rarely, never). Responses are scored 0 for the best state of health or 1 for the worst state of health. Their combinations allow different diagnostic proposals to be assigned based on summative rules. It is important to note that some labels are mutually exclusive (e.g., when the questionnaire includes both the risk diagnosis and the problem‐focused diagnosis). Each diagnostic label is or is not assigned depending on whether the sum of the scores obtained on the items related to that diagnosis exceeds a certain threshold. The questionnaire has shown good psychometric properties (construct validity, with six dimensions and 91% of the variance explained; criterion validity, with specificity ranging between 66% and 100%; strong correlations when assigning nursing diagnoses to other reference instruments; levels of agreement on test–retest reliability ranging between 56% and 91% (*p* < 0.001); and 93% internal consistency). Also, the QPSND can be both interviewer‐ and self‐administered (Brito and Aguirre 2014). The diagnostic labels assigned by the questionnaire are provisional and should be considered as preliminary hypotheses that need to be verified by a nurse during a clinical assessment interview (Brito and Aguirre 2014). After answering, respondents received a copy of their responses to the QPSND.

### 4.3 Sample Size and Statistical Power

As the prevalence of each diagnostic label in the study population was unknown, a sample size of 160 participants was estimated to be required for a 50% prevalence, a ±5% level of accuracy and 95% confidence levels, adjusting for a finite population of 250.

### 4.4 Population Sampling and Recruitment

The study population was people with ME/CFS who were members of a patient association located in Madrid (Spain). The association includes 211 people who have reported being diagnosed with ME/CFS. The inclusion criteria were being 18 years of age or older, having a medical diagnosis of ME/CFS and being an active member of the participating patient association. There were no exclusion criteria. The absence of a comprehensive list of affected individuals or an alternative sampling frame necessitated the use of a non‐probability convenience sampling method.

### 4.5 Data Sources/Collection

Respondents' answers were used as the data source. Data collection took place via an ad hoc form administered remotely (online) between May and July 2022. A paper version of the form was also produced to facilitate its completion by those with health issues that would prevent them from using electronic devices. The patient association sent an email to its members containing a link to further information about the study, as well as the informed consent form for participants. The invitation was sent to all members of the association, whether affected or not, to reach those affected individuals whose membership was held by a family member—a common practice in this type of organisation. After agreeing to participate, respondents gained access to the form and had 30 days to fill it in. Two periodic reminders were sent during that time in case they had not completed the form. Those who declined to participate were asked to answer five questions on basic sociodemographic data and reasons for non‐participation.

The form comprised three sections: sociodemographic characteristics, clinical characteristics, and psychosocial nursing diagnostic labels.

#### 4.5.1 Sociodemographic Characteristics

Data on basic sociodemographic variables were collected (age, sex, marital status, level of education, and employment status). A question on any officially recognised disability and/or dependency and the degree of disability and/or dependency was also included in this section.

#### 4.5.2 Clinical Characteristics

Clinical variables included the date of the medical diagnosis of ME/CFS and a list of 20 health problems, including comorbidities, identified in a previous study with a Spanish population living with ME/CFS (Castro‐Marrero et al. 2017). This list was supplemented by a diagnosis of coronavirus disease (COVID‐19) and/or a post‐COVID condition and/or persistent COVID. These data were self‐reported. The years since diagnosis and the person's age at that time were calculated based on participants' self‐reported age and the year in which they reported receiving a diagnosis of ME/CFS.

As mentioned above, symptoms experienced in the previous 6 months were collected using a Spanish version of the DePaul Symptom Questionnaire—Short Form (DSQ‐SF) (Jason and Coffin 2022) The Questionnaire for Psychosocial Nursing Diagnosis (QPSND) was used in the identification of potential psychosocial nursing diagnostic labels (Brito and Aguirre 2014).

### 4.6 Data Analysis

A descriptive analysis of the sociodemographic and clinical variables as well as the diagnostic labels assigned by the QPSND was performed. Continuous and discrete quantitative variables were expressed as means and standard deviations or medians and interquartile ranges depending on their distribution, and qualitative variables were expressed as absolute frequencies and relative frequencies in percentages. The normality of numerical scale variables was evaluated using the Shapiro–Wilk test and visually inspected through Quantile–Quantile (Q–Q) plots.

The relationship between the diagnostic labels was addressed using a multiple correspondence analysis (MCA). In short, a MCA is a multivariate analysis which, by means of data ordering and reduction techniques, allows multidimensional data sets to be represented in a smaller dimensional space, thus facilitating the identification of relationships between multiple categorical dependent variables (Abdi and Valentin 2007) which, in this case, are the labels assigned by the QPSND.

To this end, an MCA constructs new variables (called dimensions or axes) that seek to capture the variability observed in the data set in a smaller number of axes. Two types of graphs are typically used to present the results of this type of model: contribution bar plots and two‐dimensional plots (biplots). Contribution bar plots are bar charts representing the percentage of the variability of the model that is captured by each dimension. In biplots, however, variables and/or individuals are represented on the identified axes. Coordinates are assigned to each variable and each individual. When the responses (variables) point to the same side of the axis and are close to each other, it means that these responses tend to cluster together. In other words, individuals who answer affirmatively to one of the variables are more likely to answer affirmatively to the others. Negative responses are located in opposite quadrants, resulting in similar response profiles being clustered together.

Two key aspects must be considered when interpreting and analysing the results of an MCA. The first one is the contribution of a variable to the definition of an axis, which is expressed as a percentage. The second aspect is quality (cos2). The further a variable is located from the centre of the graph towards an axis, the better represented a variable is on that axis. Those close to the centre will therefore be poorly represented.

In this study, the active matrix is made up of 28 (dichotomous) diagnostic labels. Once the groupings have been made and identified, the supplementary variables are plotted on the graph. These variables do not alter the graph constructed, but their representation on the graph allows the different response profiles—established from the active matrix—to be related to other qualitative (sociodemographic, clinical, etc.) and quantitative variables (age or time elapsed since diagnosis). Supplementary variables will be grouped into quadrants based on their positive or negative relationship with the diagnostic label groups identified in the biplot.

The analysis was further supplemented by a hierarchical cluster analysis of the MCA results to identify clusters of individuals with similar response profiles (assigned diagnostic labels). Once the groups were established, their sociodemographic and clinical characteristics were described and compared. As there were more than two unrelated small groups, comparisons were made using non‐parametric techniques (Kruskal–Wallis tests for quantitative variables) and exact methods (Fisher's tests for comparison of proportions) depending on the type of variable. Estimates were provided with their corresponding 95% confidence interval. The employment status variables were regrouped into three categories for analysis (unemployed/unpaid domestic work; employed/retired; and on permanent/temporal sick leave) to avoid categories with too few observations.

All analyses were performed using a variety of R software packages (R, version 4.0.5, 2021‐03‐31). Specifically, the MCA was performed using the FactoMineR and factoextra software packages.

### 4.7 Ethical Considerations

The study was approved by the Research Ethics Committee for University X (approval number: 3011202123321). A briefing session was held for members of the patient association to explain the study aims in detail, what their participation would consist of, and to address any questions that they might have. All participants gave their written consent to take part in the study. The principles of the Declaration of Helsinki for medical research (World Medical Association 2013) were adopted. Participant data were handled in compliance with current Spanish data protection legislation (Ley Orgánica 3/2018 2018).

### 4.8 Quality Appraisal

This study has been reported following the Strengthening the Reporting of Observational Studies in Epidemiology (STROBE) checklist for cross‐sectional studies.

## 5 Results

**FIGURE 1:**
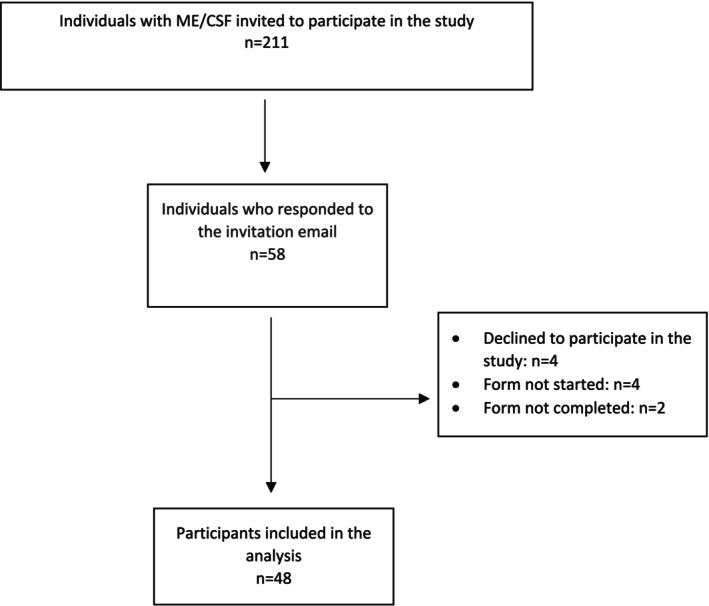
STROBE flow chart.

Fifty‐eight members of the association responded to the invitation email. Four declined, citing ‘not having enough energy at the moment’ as the most common reason. Another four did not complete the questionnaire. Ultimately, 50 participants filled in the first three sections of the form and 48 filled in the entire form: 47 completed the online version and one completed the paper version (Figure 1). The participation rate of those who were listed as living with ME/CFS in the association's records was 22.7%.

### 5.1 Subjects: Sociodemographic Characteristics of Participants

The main sociodemographic characteristics of the participants are shown in Table 1. Their mean age was 52.5 years (SD = 6.81), 95.83% were women (*n* = 46), 56.25% were married or in a civil partnership (*n* = 27), and 70.83% had a university education (*n* = 34). More than one third of the participants were actively working at the time of the study; 66.67% (*n* = 32) had an officially recognised degree of disability; and 16.67% (*n* = 8) had an officially recognised degree of dependency. Among the former, slightly more than half had an officially recognised degree of disability ranging from 25% to 49%. Among the latter, six of the eight participants reported a grade II (severe) or III (high) level of dependency.

**TABLE 1:** Sociodemographic characteristics of the respondents to the survey (*N* = 48).

Characteristics	*N* (%)	[95% CI]
Sex		
Men	2 (4.17%)	[0.72%, 15.4%]
Women	46 (95.83%)	[84.6%, 99.3%]
Age (years)		(50; 54)
Mean (SD)	52.5 (6.81)	
Median (25%–75%)	52 (47–58)	
Civil status		
Married or civil partnership	27 (56.25%)	[41.3%, 70.2%]
Separated or divorced	9 (18.75%)	[9.44%, 33.1%]
Single	8 (16.67%)	[7.97%, 30.8%]
Widowed	3 (6.25%)	[1.63%, 18.2%]
I do not wish to answer	1 (2.08%)	[0.11%, 12.5%]
Studies		
Primary school	3 (6.25%)	[1.63%, 18.2%]
High school/vocational training	10 (20.83%)	[11.0%, 35.4%]
University	34 (70.83%)	[55.7%, 82.6%]
I do not wish to answer	1 (2.08%)	[0.11%, 12.5%]
I live alone	12 (25.00%)	[14.1%, 39.9%]
Employment status		
Unemployed	8 (16.67%)	[7.97%, 30.8%]
Employed	17 (35.42%)	[22.6%, 50.6%]
Retired	11 (22.92%)	[12.5%, 37.7%]
Temporal or permanent sick leave	10 (20.83%)	[11.0%, 35.4%]
Unpaid domestic work	1 (2.08%)	[0.11%, 12.5%]
I do not wish to answer	1 (2.08%)	[0.11%, 12.5%]
I have a recognised degree of disability	32 (66.67%)	[51.5%, 79.2%]
Officially recognised degree of disability		
Class 2 1%–24%	2 (6.25%)	[1.09%, 22.2%]
Class 3 25%–49%	17 (53.13%)	[35.0%, 70.5%]
Class 4 50%–75%	13 (40.63%)	[24.2%, 59.2%]
Class 5 ≥ 75%	0 (0.00%)	[0.00%, 13.3%]
I have a recognised degree of dependency	8 (16.67%)	[7.97%, 30.8%]
Officially recognised degree of dependency		
Grade I	2 (25.00%)	[4.45%, 64.4%]
Grade II	4 (50.00%)	[21.5%, 78.5%]
Grade III	2 (25.00%)	[4.45%, 64.4%]
Year of diagnosis of ME/CFS		
(1980, 2000]	1 (2.08%)	[0.11%, 12.5%]
(2000, 2005]	5 (10.42%)	[3.90%, 23.4%]
(2005, 2010]	8 (16.67%)	[7.97%, 30.8%]
(2010, 2015]	12 (25.00%)	[14.1%, 39.9%]
(2015, 2020]	18 (37.50%)	[24.3%, 52.7%]
(2020, 2025]	4 (8.33%)	[2.70%, 20.9%]
Years from diagnosis date to 2022		(6.5; 10)
Mean (SD)	8.5 (6.73)	
Median (25%–75%)	7 (4–12)	
Age at diagnosis (estimated from years from diagnosis)		(41; 46)
Mean (SD)	44 (8.65)	
Median (25%–75%)	43.5 (38–51)	

Abbreviation: CI, confidence interval.

The mean number of years since diagnosis was 8.5 years (SD = 6.73), while the mean age at diagnosis was 44 years (SD = 8.65). Table 2 shows the frequency of health issues that participants reported having been diagnosed with in the past or currently diagnosed with. The most frequent were vitamin D deficiency (*n* = 36, 75.00%), fibromyalgia (*n* = 31, 64.58%) and anxiety disorder (*n* = 22, 45.83%).

**TABLE 2:** Prevalence of self‐reported current or past health issues among respondents (*N* = 48).

Characteristics	*N* (%)	[95% CI]
Vitamin D deficiency	36 (75.00%)	[60.11%–85.89%]
Fibromyalgia	31 (64.58%)	[49.40%–77.45%]
Anxiety disorder	22 (45.83%)	[31.64%–60.69%]
Multiple chemical sensitivity	19 (39.58%)	[26.12%–54.71%]
Shoulder tendinopathy	19 (39.58%)	[26.12%–54.71%]
Depressive disorder	19 (39.58%)	[26.12%–54.71%]
Degenerative or mechanical disease of the spine	16 (33.33%)	[20.81%–48.51%]
Hypercholesterolemia	16 (33.33%)	[20.81%–48.51%]
Hypothyroidism/Hashimoto's thyroiditis	16 (33.33%)	[20.81%–48.51%]
Ligamentous hyperlaxity	15 (31.25%)	[19.09%–46.40%]
Sjögren's syndrome or Sicca syndrome	14 (29.17%)	[17.40%–44.26%]
Plantar fasciitis	10 (20.83%)	[10.96%–35.40%]
Endometriosis	9 (18.75%)	[9.44%–33.10%]
Myofascial pain syndrome	8 (16.67%)	[7.96%–30.76%]
Carpal tunnel syndrome	6 (12.50%)	[5.19%–25.94%]
COVID‐19 infection	6 (12.50%)	[5.19%–25.94%]
Personality disorder	3 (6.25%)	[1.63%–18.21%]
Epicondylitis	3 (6.25%)	[1.63%–18.21%]
Persistent COVID or Post COVID‐19 condition	2 (4.17%)	[0.72%–15.43%]
Panic disorder	2 (4.17%)	[0.72%–15.43%]
None of them	2 (4.17%)	[0.72%–15.43%]

Abbreviation: CI, Wilson score confidence interval.

Regarding the results of the DSQ‐SF, more than 40% of participants reported experiencing 13 of the 14 symptoms all or most of the time (Table 3). More specifically, in terms of physical symptoms, 91.7% experienced *fatigue or extreme tiredness* and 87.5% experienced *next‐day soreness or fatigue after non‐strenuous, everyday activities*; in terms of cognitive symptoms, 89.6% reported *difficulty paying attention for a long period of time*. In relation to the severity of their symptoms, eight of the 14 symptoms contained in the DSQ‐SF were rated as severe or very severe by more than half of the participants. *Minimum exercise makes you physically tired* and *next‐day soreness or fatigue after non‐strenuous, everyday activities* were rated as severe or very severe by most of the subjects. A higher proportion of them reported symptoms associated with cognitive functioning, such as *difficulty paying attention for a long period of time*, to be severe or very severe.

**TABLE 3:** Results of DePaul symptom questionnaire‐short form (DSQ‐SF). Frequency and severity of the symptoms among respondents^a^ ^,^ ^b^ (*N* = 48).

Symptom	Frequency (%)					Severity (%)				
	0	1	2	3	4	0	1	2	3	4
Fatigue/extreme tiredness	0.0	4.2	4.2	39.6	52.1	0.0	4.2	22.9	45.8	27.1
Next day soreness or fatigue after non‐strenuous, everyday activities	0.0	4.2	8.3	29.2	58.3	0.0	4.2	18.8	45.8	31.2
Minimum exercise makes you physically tired	0.0	4.2	12.5	25.0	58.3	0.0	4.2	16.7	33.3	45.8
Feeling unrefreshed after you wake up in the morning	8.3	2.1	6.2	18.8	64.6	2.1	4.2	25.0	31.2	37.5
Pain or aching in your muscles	2.1	10.4	12.5	25.0	50.0	2.1	14.6	18.8	47.9	16.7
Bloating	14.6	12.5	27.1	31.2	14.6	14.6	12.5	43.8	20.8	8.3
Problems remembering things	4.2	8.3	18.8	39.6	29.2	6.2	8.3	29.2	43.8	12.5
Difficulty paying attention for a long period of time	0.0	6.2	4.2	37.5	52.1	0.0	8.3	18.8	47.9	25.0
Irritable bowel problems	22.9	10.4	20.8	14.6	31.2	22.9	10.4	25.0	20.8	20.8
Feeling unsteady on your feet, like you might fall	10.4	22.9	14.6	35.4	16.7	12.5	20.8	20.8	35.4	10.4
Cold limbs (e.g., arms, legs, hands)	8.3	16.7	14.6	31.2	29.2	10.4	16.7	31.2	22.9	18.8
Feeling hot or cold for no reason	6.2	10.4	20.8	31.2	31.2	8.3	10.4	31.2	31.2	18.8
Flu‐like symptoms	12.5	14.6	25.0	31.2	16.7	12.5	16.7	25.0	37.5	8.3
Some smells, foods, medications or chemicals make you feel sick	25.0	18.8	16.7	22.9	16.7	25.0	18.8	22.9	18.8	14.6

^a^ % of participants choosing each level of the Likert scale.

^b^ The colour gradient is continuous, increasing in intensity based on the percentage in each cell (from 0% to 100%).

Based on the QPSND responses, the most frequently identified psychosocial diagnostic label was Powerlessness, observed in more than three‐quarters of the participants (*n* = 38). Ineffective Coping (*n* = 30) and Fear (*n* = 30) were identified in more than half of the participants. Among diagnoses related to the social sphere, Social Isolation (*n* = 28) and Loneliness (*n* = 23) were reported in nearly half of the participants, while Risk for Loneliness (*n* = 20) was also a common finding (Table 4).

**TABLE 4:** Frequencies of psychosocial diagnostic labels according to questionnaire for psychosocial nursing diagnosis (QPSND) (*N* = 48).

Characteristics	*N* (%)	[95% CI]
14. Powerlessness	38 (79.17%)	[64.60%–89.04%]
6. Ineffective coping	30 (62.50%)	[47.33%–75.68%]
11. Fear	30 (62.50%)	[47.33%–75.68%]
7. Social isolation	28 (58.33%)	[43.28%–72.07%]
19. Loneliness	23 (47.92%)	[33.52%–62.64%]
21. Stress‐anxiety syndrome	23 (47.92%)	[33.52%–62.64%]
18. Chronic low self‐esteem	20 (41.67%)	[27.93%–56.72%]
20. Risk for loneliness	20 (41.67%)	[27.93%–56.72%]
1. Ineffective therapeutic regimen management	12 (25.00%)	[14.11%–39.89%]
2. Noncompliance	12 (25.00%)	[14.11%–39.89%]
12. Chronic sorrow	12 (25.00%)	[14.11%–39.89%]
17. Risk for situational low self‐esteem	12 (25.00%)	[14.11%–39.89%]
22. Caregiver role strain	12 (25.00%)	[14.11%–39.89%]
25. Grieving	12 (25.00%)	[14.11%–39.89%]
27. Risk for complicated grieving	12 (25.00%)	[14.11%–39.89%]
4. Moral distress	11 (22.92%)	[12.51%–37.67%]
9. Stress overload	11 (22.92%)	[12.51%–37.67%]
24. Caregiver diversional activity deficit	9 (18.75%)	[9.44%–33.10%]
13. Hopelessness	8 (16.67%)	[7.96%–30.76%]
15. Risk for powerlessness	7 (14.58%)	[6.54%–28.38%]
3. Spiritual distress	5 (10.42%)	[3.90%–23.44%]
26. Complicated grieving	4 (8.33%)	[2.70%–20.87%]
5. Ineffective health maintenance	3 (6.25%)	[1.63%–18.21%]
16. Situational low self‐esteem	2 (4.17%)	[0.72%–15.43%]
23. Risk for caregiver role strain	2 (4.17%)	[0.72%–15.43%]
8. Impaired social interaction	1 (2.08%)	[0.11%–12.47%]
10. Anxiety	1 (2.08%)	[0.11%–12.47%]
28. Disturbed body image	1 (2.08%)	[0.11%–12.47%]

Abbreviation: CI, Wilson score confidence interval.

Regarding the MCA, the total inertia (sum of variances) of the first two dimensions was 28%; with 18.5% of the variance explained for dimension 1 (*x*‐axis) and 9.5% for dimension 2 (*y*‐axis). However, 70% of the variance explained is only reached in dimension 9, but the tipping point was observed in dimension 2. The variables with the highest contributions to the definition of axis 1 were Chronic Sorrow (7.26%), Hopelessness (6.37%), Social Isolation (5.79%) and Stress‐Anxiety Syndrome (5.33%) (Figure S1). On the other hand, the following stand out in the definition of axis 2: Risk for Complicated Grieving (13.32%), Risk for Powerlessness (10.93%), Powerlessness (8.33%) and Risk for Loneliness (6.5%) (Figure S2). Of these, 17 variables were significantly associated with dimension 1: Decreased Diversional Activity Engagement (in caregivers), Moral Distress, Caregiver Role Strain, Risk for Powerlessness, Stress Overload, Complicated Grieving, Chronic Low Self‐Esteem, Ineffective Coping, Spiritual Distress, Risk for Loneliness, Powerlessness, Fear, Hopelessness, Loneliness, Chronic Sorrow, Social Isolation and Stress‐Anxiety Syndrome. In the case of dimension 2, the variables that were significantly associated were: Noncompliance, Grieving, Ineffective Coping, Risk for Situational Low Self‐Esteem, Powerlessness, Risk for Loneliness, Loneliness, Risk for Powerlessness and Risk for Complicated Grieving (Figure S2).

**FIGURE 2:**
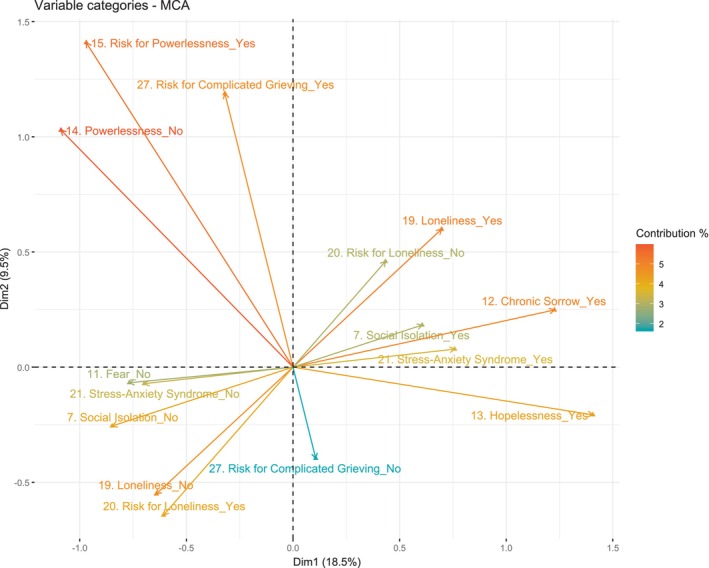
Biplot—MCA. Relationship patterns among psychosocial diagnostic labels according to the QPSND.

Figure 2 shows the projection of the 15 variables with the highest contributions (colour scale) and best represented (cos2 > 0.3). As can be observed, axis 1 seems to clearly separate at least two groups of people. Subjects with more unfavourable profiles are grouped on the positive side, based on the psychosocial diagnostic labels they were assigned: Loneliness, Chronic Sorrow, Hopelessness, Stress‐Anxiety Syndrome, and Social Isolation. Risk for Powerlessness, Risk for Complicated Grieving and Risk for Loneliness are grouped on the negative scale of the axis, along with the negative versions of the ones on the right‐hand side.

**FIGURE 3:**
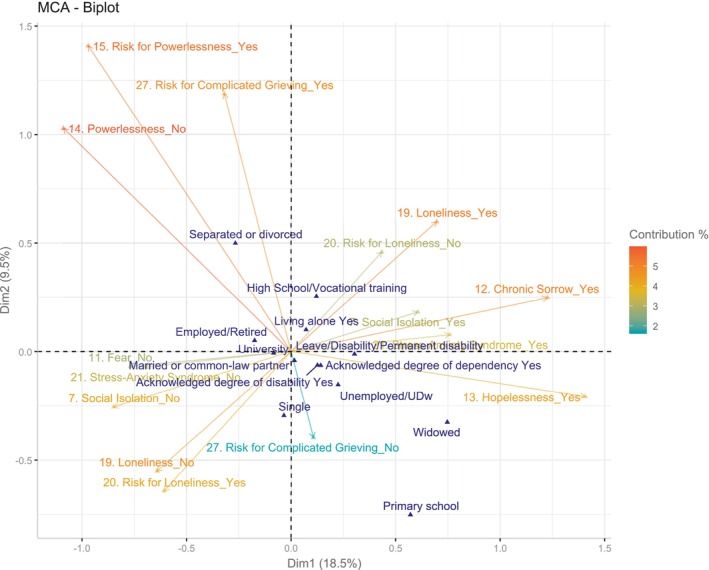
Biplot—MCA. Multivariate associations between psychosocial diagnostic labels and qualitative supplementary sociodemographic variables.

**FIGURE 4:**
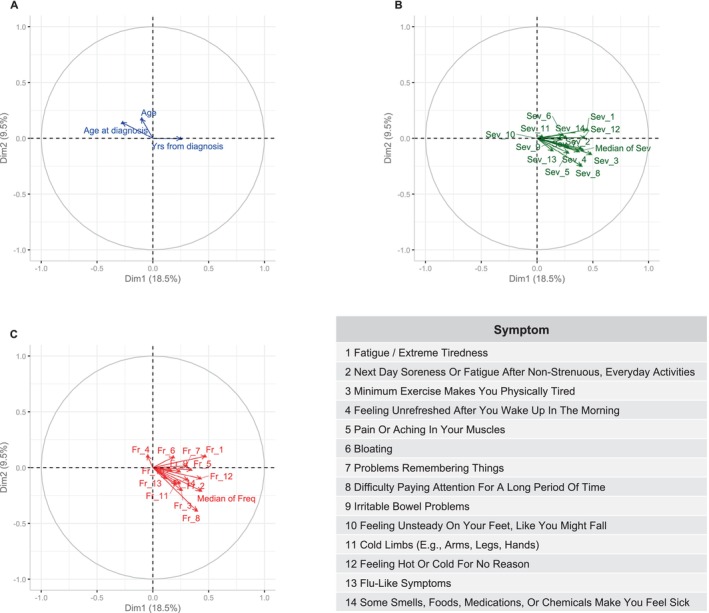
Biplot—MCA. Multivariate associations between psychosocial diagnostic labels and quantitative supplementary variables.

When the supplementary qualitative sociodemographic variables are included in the biplot (Figure 3), characteristics such as being a widow/widower, having primary education, or being on a temporary/permanent sick leave seem to correlate with subjects with poorer diagnostic profiles, although the relationship of each of these variables with the axes means the null hypothesis cannot be rejected. Regarding quantitative variables, a longer time since diagnosis was associated with a poorer diagnostic profile. Being negatively correlated with the age at diagnosis (Spearman's *ρ* = −0.6 [−0.75; −0.39]), being older at diagnosis was associated with a better profile (Figure 4A).

Regarding the DSQ‐SF results, a higher severity of a number of symptoms—*physical tiredness after minimum exercise*, *fatigue or extreme tiredness*, *feeling hot or cold for no reason*, *difficulty paying attention for a long period of time*, *next‐day soreness or fatigue after non‐strenuous, everyday activities* and *problems remembering things* was positively associated with the first dimension, that is, to a poorer profile (Figure 4B). The same is true for their frequency (Figure 4C). Taking all symptoms into consideration, higher median frequency and severity scores are associated with what we have termed a poorer psychosocial diagnostic profile.

When health issues were mapped onto the biplot as supplementary qualitative variables, it could be observed that myofascial pain syndrome, sjögren's syndrome, depressive disorder, fibromyalgia, anxiety disorder and multiple chemical sensitivity all appear on the positive side of axis 1 in a statistically significant manner, while hypercholesterolaemia, endometriosis and panic disorder appear on the negative side of the first axis, although not in a statistically significant manner (Figure S3).

**FIGURE 5:**
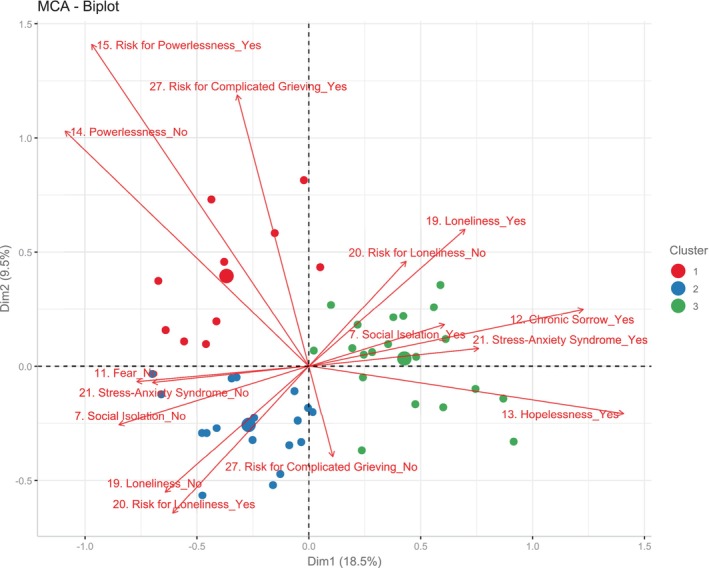
Biplot—MCA. Patient representation based on cluster assignments derived from the multivariate structure of psychosocial diagnostic labels.

The cluster analysis of the active matrix constructed from the psychosocial diagnoses identified three clearly differentiated groups (Figure 5). Axis 1 clearly separates cluster 3 from clusters 1 and 2, while axis 2 separates the latter two. Table 5 describes and compares their sociodemographic characteristics and Table 6 describes their differences in terms of health issues. Looking at both tables, it is apparent that cluster 1 includes highly educated, employed or retired individuals who are not living alone, have lower levels of dependency and disability, and for whom less time has passed since being diagnosed than the individuals in clusters 2 and 3. Cluster 2 has the highest proportion of university graduates. They are mostly employed or retired, do not live alone and have a higher percentage of officially recognised dependency or disability than those in cluster 1. They were younger at the time of diagnosis than those in cluster 1 and have a median degree of severity of symptoms, similar to that of cluster 1.

**TABLE 5:** Description of sociodemographic characteristics by cluster according to MCA analysis.

Characteristics	Overall, *N* = 48^a^	Cluster 1, *N* = 10^a^	Cluster 2, *N* = 18^a^	Cluster 3, *N* = 20^a^	*p* ^b^
Sex					
Men	2 (4.17%)	1 (10%)	0 (0%)	1 (5%)	0.681
Women	46 (95.83%)	9 (90%)	18 (100%)	19 (95%)	
Age (years)					
Mean (SD)	52.5 (6.8)	55.8 (8.7)	51.1 (5.4)	52.1 (6.7)	0.113
Median (25%–75%)	52 (47–58)	58.5 (53.3–60.8)	51.5 (47.8–54.8)	53 (46.8–58)	
Civil status					
Married or civil partnership	27 (56.25%)	5 (50%)	9 (50%)	13 (65%)	0.626
Separated or divorced	9 (18.75%)	4 (40%)	3 (16.67%)	2 (10%)	
Single	8 (16.67%)	1 (10%)	4 (22.22%)	3 (15%)	
Widowed	3 (6.25%)	0 (0%)	1 (5.55%)	2 (10%)	
I do not wish to answer	1 (2.08%)	0 (0%)	1 (5.55%)	0 (0%)	
Studies					
Primary school	3 (6.25%)	0 (0%)	1 (5.55%)	2 (10%)	0.666
High school/vocational training	10 (20.83%)	3 (30%)	2 (11.11%)	5 (25%)	
University	34 (70.83%)	7 (70%)	15 (83.33%)	12 (60%)	
I do not wish to answer	1 (2.08%)	0 (0%)	0 (0%)	1 (5%)	
Employment status					
Employed/retired	28 (58.33%)	7 (70%)	13 (72%)	8 (40%)	0.430
Unemployed/unpaid domestic work	9 (19.15%)	1 (10%)	2 (11%)	6 (30%)	
Temporal or permanent sick leave	10 (20.83%)	2 (20%)	3 (17%)	5 (25%)	
I do not wish to answer	1 (2.08%)	0 (0%)	0 (0%)	1 (5.0%)	
I live alone	12 (25%)	3 (30%)	5 (27.78%)	4 (20%)	0.756
I have a recognised degree of disability	32 (66.67%)	5 (50%)	11 (61%)	16 (80%)	0.234
I have a recognised degree of dependency	8 (16.67%)	1 (10%)	3 (16.67%)	4 (20%)	0.889
Years from diagnosis (Date to 2022)	7.0 (4.0–12.0)	5.5 (5.0–8.8)	7.0 (3.3–12.0)	7.5 (4.8–13.3)	0.767
Age at diagnosis (estimated from years from diagnosis)	44 (38–51)	51 (45–55)	44 (39–49)	41 (37–51)	0.114
Median of severity of symptoms [Likert 1–5]	3.00 (2.00–3.00)	2.00 (1.25–3.00)	2.25 (2.00–3.00)	3.00 (2.50–3.13)	0.029
Median of frequency of symptoms [Likert 1–5]	3.00 (2.50–3.50)	2.25 (2.00–3.00)	3.00 (2.13–3.00)	3.25 (3.00–3.63)	0.026

^a^ *n* (%); mean (SD); median (25%–75%).

^b^ Fisher's exact test; Kruskal–Wallis rank sum test.

**TABLE 6:** Description of health issues by cluster according to MCA analysis.

Characteristics	Overall, *N* = 48^a^	Cluster 1, *N* = 10^a^	Cluster 2, *N* = 18^a^	Cluster 3, *N* = 20^a^	*p* ^b^
Vitamin D deficiency	36 (75%)	5 (50%)	14 (77.77%)	17 (85%)	0.146
Fibromyalgia	31 (64.58%)	6 (60%)	7 (38.88%)	18 (90%)	0.003
Anxiety disorder	22 (45.83%)	4 (40%)	5 (27.77%)	13 (65%)	0.071
Multiple chemical sensitivity	19 (39.58%)	4 (40%)	4 (22.22%)	11 (55%)	0.129
Shoulder tendinopathy	19 (39.58%)	6 (60%)	5 (27.77%)	8 (40%)	0.260
Depressive disorder	19 (39.58%)	3 (30%)	5 (27.77%)	11 (55%)	0.207
Degenerative or mechanical disease of the spine	16 (33.33%)	2 (20%)	5 (27.77%)	9 (45%)	0.354
Hypercholesterolemia	16 (33.33%)	1 (10%)	8 (44.44%)	7 (35%)	0.217
Hypothyroidism/Hashimoto's thyroiditis	16 (33.33%)	3 (30%)	6 (33.33%)	7 (35%)	> 0.999
Ligamentous hyperlaxity	15 (31.25%)	2 (20%)	6 (33.33%)	7 (35%)	0.784
Sjögren's syndrome or Sicca syndrome	14 (29.17%)	1 (10%)	2 (11.11%)	11 (55%)	0.005
Plantar fasciitis	10 (20.83%)	2 (20%)	2 (11.11%)	6 (30%)	0.379
Endometriosis	9 (18.75%)	2 (20%)	4 (22.22%)	3 (15%)	0.894
Myofascial pain syndrome	8 (16.67%)	0 (0%)	2 (11.11%)	6 (30%)	0.089
Carpal tunnel syndrome	6 (12.50%)	1 (10%)	2 (11.11%)	3 (15%)	> 0.999
COVID‐19 infection	6 (12.50%)	1 (10%)	4 (22.22%)	1 (5%)	0.283
Personality disorder	3 (6.25%)	0 (0%)	1 (5.55%)	2 (10%)	0.792
Epicondylitis	3 (6.25%)	1 (10%)	1 (5.55%)	1 (5%)	> 0.999
Persistent COVID or post COVID‐19 condition	2 (4.17%)	0 (0%)	1 (5.55%)	1 (5%)	> 0.999
Panic disorder	2 (4.17%)	1 (10%)	0 (0%)	1 (5%)	0.681
None of them	2 (4.17%)	1 (10%)	1 (5.55%)	0 (0%)	0.335

^a^ *n* (%).

^b^ Fisher's exact test.

Cluster 3 has a higher proportion of people with primary and secondary education than clusters 1 and 2, a higher percentage of married and widowed individuals and—although the years since diagnosis are close to those of cluster 2—they were diagnosed at a younger age and have a higher median degree of severity and frequency of symptoms than the other two clusters. Compared to the other two clusters, they reported a higher proportion of diagnoses of fibromyalgia (90%), vitamin D deficiency (85%), anxiety disorder (65%), depressive disorder (55%), multiple chemical sensitivity (55%), and Sjögren's syndrome (55%), among others, with these differences being statistically significant for fibromyalgia (*p* = 0.003) and Sjögren's syndrome (*p*‐value = 0.005). Thus, this group has a poorer psychosocial diagnostic profile, which is related to a sociodemographic profile that is less educated, has a longer duration of illness, and experiences symptoms with greater frequency and severity, as well as most of the health problems analysed with the same or greater frequency.

## 6 Discussion

Study participants living with ME/CFS exhibit a high prevalence of psychosocial nursing diagnoses. The literature on the prevalence of such diagnoses in specific populations is both limited and outdated, which should be considered when assessing the scope and reliability of direct comparisons with earlier studies. Nonetheless, comparisons with findings from other studies can still yield valuable insights. The percentage of people assigned the label Powerlessness (79.17%) is much higher than the most common label in other studies that have used the QPSND as a diagnosis assignment tool. In these studies in particular, the most frequently assigned labels to participants did not exceed 45%, these being Fear (44%) (Brito and Aguirre 2014) and Stress‐Anxiety Syndrome, which in this case was present in 41.5% of the sample (Sanz Sánchez 2017). The higher psychosocial distress in our study participants could be explained by the high prevalence of certain characteristics—namely, a greater number of female participants; a high percentage of participants living alone; participants having a disability; and participants being medically diagnosed with anxiety‐depression—, which, according to the findings of Brito and Aguirre (2014), are associated with a higher likelihood of diagnostic label assignment by the QPSND. Differences in the specific labels assigned were also observed. Labels relating to coping responses (i.e., Powerlessness, Ineffective Coping, Fear, Stress‐Anxiety Syndrome) and social comfort (Social Isolation, Risk for Loneliness, Loneliness) are notably present in our sample. In the studies by Brito and Aguirre (2014) and Sanz Sánchez (2017), the labels describing coping responses are also common (Fear, Stress‐Anxiety Syndrome, Powerlessness), but there are also labels referring to the grieving process (Grieving), to medication self‐management (Noncompliance), and to a lesser degree, to behaviour/social interaction (Risk for Loneliness, Loneliness, Impaired Social Interaction). These different profiles, in terms of both label prevalence and sociodemographic characteristics, may be linked to the setting in which the surveys were administered. For instance, Brito and Aguirre (2014) and Sanz Sánchez (2017) collected data in primary care nursing consultations, whereas our study focused on a patient association comprising active members living with ME/CFS.

Powerlessness is the most prevalent diagnostic label in our sample. According to Miller (2000), chronically ill people may have deficits in one or more patient ‘power resources’—physical strength and reserve; psychological stamina and social support; positive self‐concept (self‐esteem); energy; knowledge; motivation and belief system (hope)—, resulting in Powerlessness. This theoretical framework provides an explanation for the study results when considering symptoms of ME/CFS, or prevalent problems observed in the participants, which would compromise several ‘power resources’ pointed out by Miller. Regarding Ineffective Coping, Braga and da Cruz (2005) point out that this diagnosis describes a human response that, in turn, can trigger other dysfunctional responses. According to these authors, it is plausible that Ineffective Coping predisposes individuals to feelings of Powerlessness. However, it would also be possible to consider the opposite direction of this relationship, whereby the sense of inadequate control that characterises Powerlessness would predispose individuals to Ineffective Coping (Herdman et al. 2021). Although the label Ineffective Coping refers solely to the outcome or product of coping rather than to coping phenomena (Becket 1991), the high prevalence of this label—despite the years elapsed since diagnosis (median = 7 years)—suggests the complexity of the disease coping process. In our view, effective nursing support is essential to enhance the development of adaptive strategies to cope with the disease and, at the same time, to increase the sense of control, a loss of which leads to Powerlessness.

In line with previous research, it stands out that several of the aforementioned diagnoses appear to be linked to social interaction. This suggests that those living with the disease face a profound negative impact on their social lives and a considerable reduction in social relationships. These issues stem not only from the limitations associated with managing the disease but also from negative responses from family and friends, who may exhibit misunderstanding, confusion, or doubts about the reality of the illness and the functional loss it entails (Wotherspoon 2024).

Regarding the MCA, dimension 1 separates the labels denoting a high emotional and social burden from other labels which, a priori, refer to risk‐related diagnoses and, consequently, to less psychosocial involvement. When considering the labels that are best represented (cos2) and contribute most to axes 1 and 2, the positive side of dimension 1 would group together the labels Loneliness, Chronic Sorrow, Social Isolation, Stress‐Anxiety Syndrome and Hopelessness. To the best of our knowledge, there are no previous studies addressing the relationship between these labels in a multivariable manner, but there is evidence of a bivariate linkage between them (Brito and Aguirre 2014).

On the other hand, common responses for the diagnostic labels grouped on the positive side of the dimension 1 axis would be the consequences of the losses associated with the illness. The multiple losses experienced by people living with ME/CFS have been well documented in previous literature and are related to family and work roles, social relationships, and financial resources (Bartlett et al. 2022; Sandhu et al. 2021), often accompanied by a profound identity crisis (Dickson et al. 2008). Diagnostic labels such as Loneliness, Social Isolation, Hopelessness or Stress‐Anxiety Syndrome would label responses to these ongoing losses and the disparity between the present reality and the desired one, characteristic of Chronic Sorrow (Eakes et al. 1998).

The grouping of these labels might acquire greater significance when based on the four‐phase model proposed by Fennell (1995) and Fennell et al. (2021), which helps to understand the different phases that individuals may go through when facing ME/CFS. From a proposal based on systems theory, this author considers the individual with ME‐CFS, their family, work environment, social network and community in general as dynamic, interactive, and interdependent factors (Jason et al. 2000). Her four‐phase model of illness experience describes the physical, psychological, social and occupational functioning characteristic of each phase. Following this proposal, the human responses of Loneliness, Chronic Sorrow, Social Isolation, Hopelessness and Stress‐Anxiety Syndrome could occur in people who are in phase 3. At the psychological level, this phase is characterised by a ‘secondary emotional crisis or grief reaction’ (Fennell 1995; Fennell et al. 2021) when people with ME/CFS realise that their lives have changed, that they are not the same as they used to be and never will be again. This experience of loss extends to the social sphere, with feelings of isolation, abandonment and stigmatisation. However, as the author points out, given the changing nature of the disease, with periods of relapse and remission, people may return to a previous phase or even be in more than one phase at the same time, meaning that placing them in phase 3 would not necessarily be definitive. Beyond its explanatory value for the findings of this study, Fennell's proposal is particularly noteworthy for its potential to validate the illness experience of individuals with ME/CFS, thereby mitigating epistemic injustice (Byskov 2020) and alleviating the associated suffering.

Continuing with the MCA, and in relation to the labels grouped on the negative side of dimensions 1 and 2 (Risk for Loneliness, Risk for Powerlessness and Risk for Complicated Grief), these seem to be a mirror image of those on the positive side—albeit describing situations of risk, which stands to reason when considering the questionnaire's incompatibility rules for assigning labels. In any case, the relevance of this grouping lies in the fact that it would be signalling a susceptibility to develop certain human responses (Powerlessness, Complicated Grief or Loneliness) given the presence of risk factors, and according to the notion of risk diagnosis as defined by the NANDA‐I Classification of Nursing Diagnoses (Herdman et al. 2021). In short, the distribution of diagnostic labels across the different axes would account for the varying experiences of people with ME/CFS (Fennell et al. 2021) and, therefore, for the value of nursing diagnoses in capturing the diversity of human responses to the same clinical condition.

The projection of the supplementary sociodemographic variables suggests that the profiles in the labels are not independent of these variables. Although the lack of statistical power requires caution in result interpretation, certain characteristics—such as widowhood, having primary education, being on sick leave—seem to cluster in the same quadrants in which the diagnostic labels describing a greater psychosocial involvement are located. Being employed or retired, having a university education, and being divorced or separated are all associated with labels such as Risk for Powerlessness, Risk for Loneliness or Risk for Complicated Grief, that is, with a lower level of involvement. While, in isolation, the study by Brito and Aguirre (2014) associates certain sociodemographic characteristics with the assignment of QPSND labels, the multivariate analysis conducted in our study provides insights into the combined behaviour of several of these characteristics. An overview of the profile of the group that we have considered to be more psychosocially involved would seem to bear a certain resemblance to the characteristics of those Brito and Aguirre (2014) suspected of having psychosocial problems, such asbeing female, having a low level of education, not having a paid job, and having a physical disability. With respect to the years elapsed since diagnosis, the fact that the vector points to the side of individuals with a more adverse psychosocial profile is consistent with what has been discussed so far. As noted above, we can predict that over the course of the disease (and therefore over the years) there will be significant ongoing losses, to which people with ME/CFS would respond with Hopelessness, Chronic Sorrow, Stress‐Anxiety Syndrome or Isolation. Identifying these losses is essential to contextualise nursing interventions aimed at helping people discover and make the most of their own resources and potential, and to help them acquire other resources that are lacking or non‐existent.

The frequency and severity of the symptoms reported by our participants and collected by the DSQ‐SF is high. The patients in our sample have higher mean scores on almost all items than, for instance, Froelich et al.'s sample of the German population with a self‐reported diagnosis of ME/CFS (Froehlich et al. 2021). On the other hand, the MCA carried out in our study seems to suggest that the frequency and severity of symptoms as measured by the DSQ‐SF is also not distributed independently of the QPSND labels. Therefore, higher frequency and severity scores are associated with a more adverse psychosocial profile. These results suggest that symptom intensity goes hand‐in‐hand with human responses that highlight the sufferer's subsequent difficulties in social situations and in successfully adapting to the disease. A high frequency and severity of core ME/CFS symptoms, such as fatigue, post‐exertional malaise, pain and neurocognitive dysfunction, can be expected to be associated with greater psychosocial involvement. Feeling hot or cold for no apparent reason, which is a neuroendocrine manifestation, also seems to be related to this profile, despite not being one of the most prevalent symptoms in the ME/CFS population (Castro‐Marrero et al. 2017).

As with symptoms, certain health problems also appear to be related to QPSND label profiles. Far from being randomly distributed, the most adverse psychosocial profile would seem to identify people with a clinical profile with associated health problems such as Sjögren's syndrome, depressive disorder, fibromyalgia, anxiety, multiple chemical sensitivity and myofascial pain syndrome. Some of these results are in line with the bivariate relationships identified by Brito and Aguirre (2014). According to these authors, having a psychological diagnosis of anxiety or depression was associated with the assignment by the QPSND of the diagnostic labels of Loneliness, Chronic Sorrow, Social Isolation and Stress‐Anxiety Syndrome, among others, which, in our study, would describe most of the human responses present in people with greater psychosocial involvement. Our research included other health problems that seem to be associated with this most adverse profile, such as Sjögren's syndrome, fibromyalgia, multiple chemical sensitivity and myofascial pain syndrome.

The cluster analysis confirms and facilitates the creation of the subgroups that were hinted at in the MCA on which it is based. The three identified clusters reveal two groups that are similar to each other (clusters 1 and 2) and a third (cluster 3) with a quite distinct sociodemographic, clinical, comorbid and psychosocial nursing diagnoses profile. Albeit with nuances, cluster 3 would share similarities with subgroups 1 and 2, identified by Castro‐Marrero et al. (2017) in their cluster analysis based on clinical variables. In their study, subgroups 1 and 2 once again have the poorest scores for fatigue and quality of life, whereas in our study, cluster 3 has the highest median levels of symptom severity and frequency of symptoms. Also, the health issues in subgroups 1 and 2 and cluster 3 are similar, as they cluster the highest prevalence rates—compared to the other subgroups—of fibromyalgia, myofascial pain syndrome, sicca syndrome, degenerative or mechanical spinal disease, plantar fasciitis, multiple chemical sensitivity and anxiety disorder. Nevertheless, there are differences in the percentages of people experiencing the various clinical conditions in the two studies. The lack of clear correspondence between the subgroups of the two studies could be explained by differences in the participating populations (patients seen at a specialised ME/CFS unit vs. members of a patient association living with ME/CFS) and the data collection methods (clinical records vs. self‐reported conditions). The cluster analysis shows that the poorest diagnostic profile is statistically associated with the highest level of symptom intensity, both in terms of frequency and severity, and with self‐reported clinical conditions that often co‐occur with ME/CFS, such as fibromyalgia and Sjögren's syndrome. This supports previous evidence for the existence of subgroups within the ME/CFS population based not only on their comorbidities but also on their diagnostic profiles, thus differentiating populations at risk of presenting certain nursing diagnoses (as is the case among those grouped in clusters 1 and 2 of our study) and others in which such diagnoses are already present. At the same time, it also shows that poorer symptom profiles and greater psychosocial impairment tend to cluster together (cluster 3). The possibility of using tools that can be easily incorporated into daily clinical practice to differentiate between several profiles of individuals living with ME/CFS that are associated with psychosocial nursing diagnoses can be very useful for nursing professionals. This is because these tools both facilitate the identification of said profiles and diagnoses and, as a result, also the anticipation of interventions and subsequent referral to other professionals when necessary (Brito and Aguirre 2014).

### 6.1 Strengths and Limitations of the Work

This is the first study to address psychosocial nursing diagnoses of people with ME/CFS using a validated diagnostic support tool. It combines the identification of psychosocial nursing diagnoses with previous cluster analyses, making it possible to characterise the different subgroups of the population living with ME/CFS, both in sociodemographic terms and clinically, while highlighting their emotional care needs and requirements for successful social functioning.

The use of a non‐probability sampling method, combined with a low participation rate, may have introduced considerable selection bias, potentially compromising the external validity of the study. Despite the mechanisms implemented at various levels to encourage participation (a dedicated session to present the study to association members, the option to complete the form either online or on paper with ample time to do so, and the use of both automatic and manual reminder strategies), largely designed to mitigate the physical and cognitive limitations imposed by the disease, it was not possible to achieve the required sample size. Given the experiences of invalidation often associated with this illness, it is possible that the study's objective discouraged participation to some extent, as psychosocial diagnoses might have been perceived as questioning the organic basis of ME/CFS (Daniels et al. 2017). In any case, participant self‐selection imposes a bias on the estimates, the direction of which depends on the factors most influential in deterring participation.

A small sample size increases the variability of estimators, reducing the actual coverage of confidence intervals, particularly for extreme proportions, even though the Wilson method was employed for their calculation. This leads to a higher probability of committing a Type I error than typically anticipated (*α*). Moreover, failing to reach the predetermined sample size reduces the statistical power initially planned, limiting the ability to detect differences between groups even when such differences genuinely exist. Additionally, using numerous categories in a multiple correspondence analysis (MCA) with a small sample may result in hyper‐parameterisation, thus complicating the reliable estimation of relationships between variables and, consequently, the identification of robust patterns. It may also lead to some degree of overfitting to the specific characteristics of the sample, thereby failing to reflect genuine relationships.

Finally, while MCA is an exploratory method rather than a fitted model in the classical sense, working with so many variables in such a limited sample could have introduced uncontrolled confounders. For these reasons, it is crucial to interpret confidence intervals and hypothesis test results with caution, even if the findings seem reasonably consistent with the conceptual hypotheses proposed.

Despite these limitations, the results obtained in our study are consistent and, although they should be confirmed in future research, they undoubtedly have considerable value for developing understanding and also clinical utility.

### 6.2 Recommendations for Further Research

The results of our study should be confirmed in larger samples of people living with ME/CFS and in populations from other epidemiological contexts. Given the greater psychosocial involvement of individuals in cluster 3, it would be advisable to implement participatory action research proposals to further explore this human experience, while designing interventions to address the psychosocial responses of this highly vulnerable subgroup.

Labelling these psychosocial issues using standardised nursing language contributes to the identification of care needs and, consequently, to informing nursing practice and the provision of appropriate care resources. From a clinical point of view, the psychosocial needs of populations with great health requirements, such as those with ME/CFS, are more clearly stated when standardised labels are used. This makes it more feasible to plan and implement effective nursing interventions based on the needs identified that will contribute to improving these patients' quality of life.

## 7 Conclusion

The illness experience of individuals with ME/CFS is characterised by high levels of distress, in which physical, emotional and social aspects intertwine to create profiles with varying degrees of involvement and response. This study proposes several characteristics that appear to be linked to poorer psychosocial profiles. It also shows how nursing diagnostic labels are aligned with the degree of severity and frequency of disease symptoms, while highlighting the importance of identifying the psychosocial needs of these individuals for the design of targeted care and treatment plans.

## Author Contributions

Conceptualisation: C.O.‐Q., A.A.‐C., P.R.B.‐B., J.E.‐H. Data curation: C.O.‐Q., J.E.‐H. Formal analysis: C.O.‐Q., A.A.‐C., V.H.‐B., J.E.‐H. Funding acquisition: C.O.‐Q. Investigation: C.O.‐Q., A.A.‐C., J.E.‐H. Methodology: C.O.‐Q., A.A.‐C., P.R.B.‐B., A.I.P.‐M., M.T.A.‐C., T.G.‐G., V.H.‐B., J.E.‐H. Project administration: C.O.‐Q. Resources: C.O.‐Q., J.E.‐H. Software: C.O.‐Q., J.E.‐H. Supervision: A.A.‐C., J.E.‐H. Validation: P.R.B.‐B., J.E.‐H. Visualisation: C.O.‐Q., A.A.‐C., P.R.B.‐B., A.I.P.‐M., M.T.A.‐C., T.G.‐G., J.E.‐H. Writing – original draft preparation: C.O.‐Q., A.A.‐C., P.R.B.‐B., A.I.P.‐M., M.T.A.‐C., T.G.‐G., J.E.‐H. Writing – review and editing: C.O.‐Q., A.A.‐C., P.R.B.‐B., A.I.P.‐M., M.T.A.‐C., T.G.‐G., V.H.‐B., J.E.‐H.

## Conflicts of Interest

The authors declare no conflicts of interest.

## Supporting information


**Figure S1.** Bar plot showing the contribution of each psychosocial diagnostic label to Dimension 1.

**Figure S2.** Bar plot showing the contribution of each psychosocial diagnostic label to Dimension 2.

**Figure S3.** Biplot—MCA. Relationship between psychosocial diagnostic labels and self‐reported current or past health issues.

## Data Availability

The data that support the findings of this study are available from the corresponding author upon reasonable request.
