# Correction to "Psychosocial Nursing Diagnoses of Individuals With Myalgic Encephalomyelitis‐Chronic Fatigue Syndrome: A Descriptive Study"

**DOI:** 10.1002/nop2.70391

**Published:** 2025-12-19

Oter‐Quintana, C., A. Alameda‐Cuesta, P. R. Brito‐Brito et al. 2025. “Psychosocial Nursing Diagnoses of Individuals With Myalgic Encephalomyelitis‐Chronic Fatigue Syndrome: A Descriptive Study.” *Nursing Open* 12, no. 5: e70212. https://doi.org/10.1002/nop2.70212.

In Section 4.7, Ethical Considerations, where it currently says ‘University X’, it should read ‘Rey Juan Carlos University’.

In Section 5.1, Subjects: Sociodemographic Characteristics of Participants, in the paragraph 3 where it says ‘In relation to the severity of their symptoms, eight of the 14 symptoms contained in the DSQ‐SF were rated as severe or very severe by more than half of the participants ….’, it should instead say ‘In relation to the severity of their symptoms, seven of the 14 symptoms contained in the DSQ‐SF were rated as severe or very severe by more than half of the participants ….’.

We apologise for this error.

