# Supplementary figures and images for: Psychosocial Nursing Diagnoses of Individuals With Myalgic Encephalomyelitis‐Chronic Fatigue Syndrome: A Descriptive Study
Source: Nurs Open. 2025 Apr 30;12(5):e70212. doi: 10.1002/nop2.70212 (PMC12041938; doi:10.1002/nop2.70212)

### Contribution of variables to Dim-1

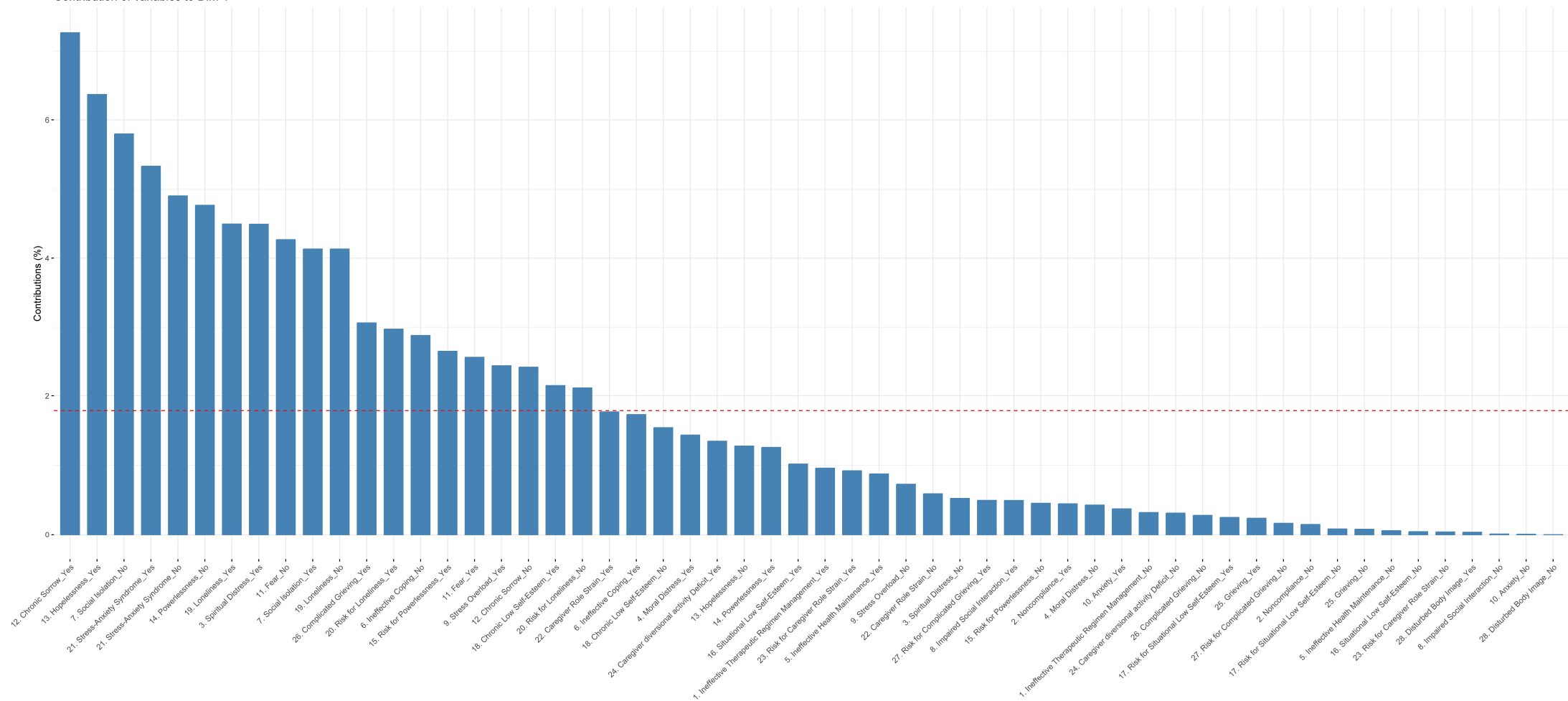

Supplement: Supplementary file 1 — Figure S1. Bar plot showing the contribution of each psychosocial diagnostic label to Dimension 1. [file NOP2-12-e70212-s003.pdf]

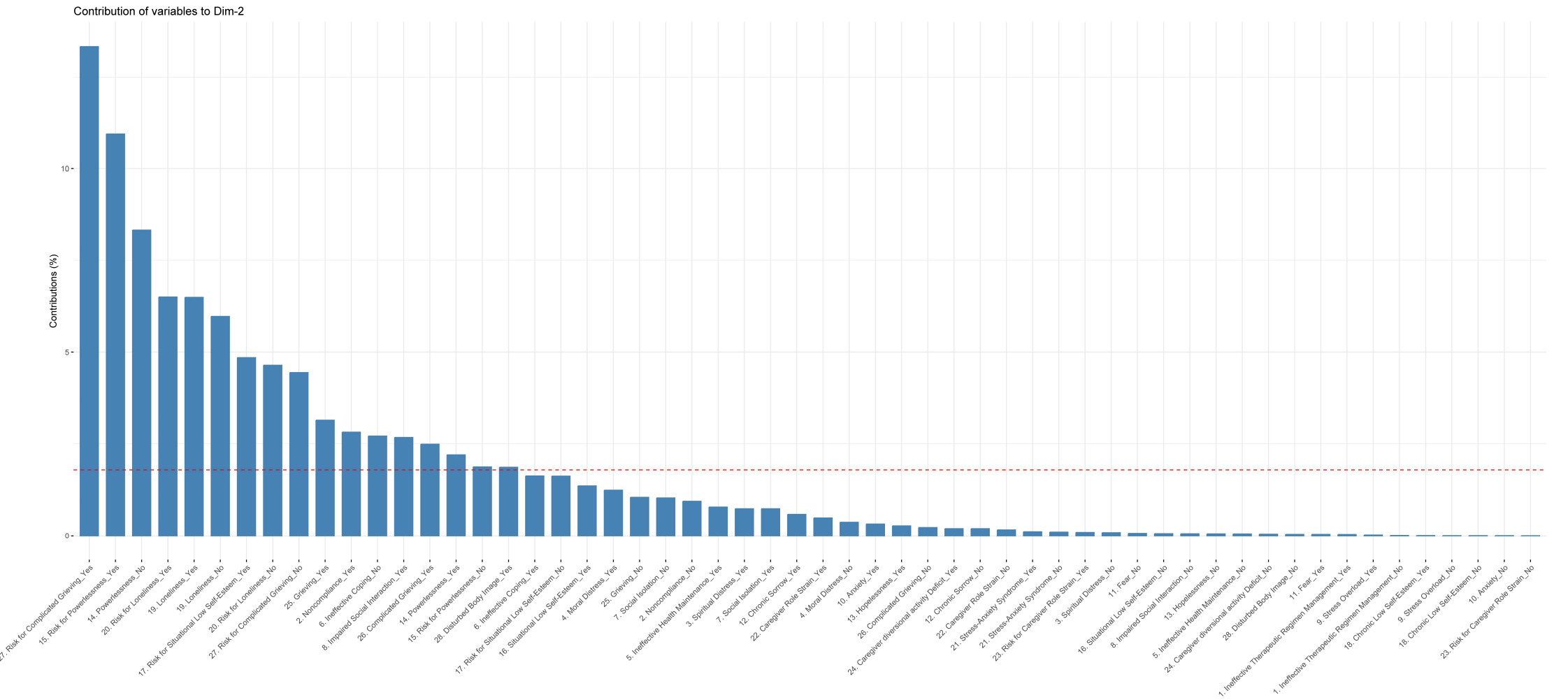

Supplement: Supplementary file 2 — Figure S2. Bar plot showing the contribution of each psychosocial diagnostic label to Dimension 2. [file NOP2-12-e70212-s001.pdf]

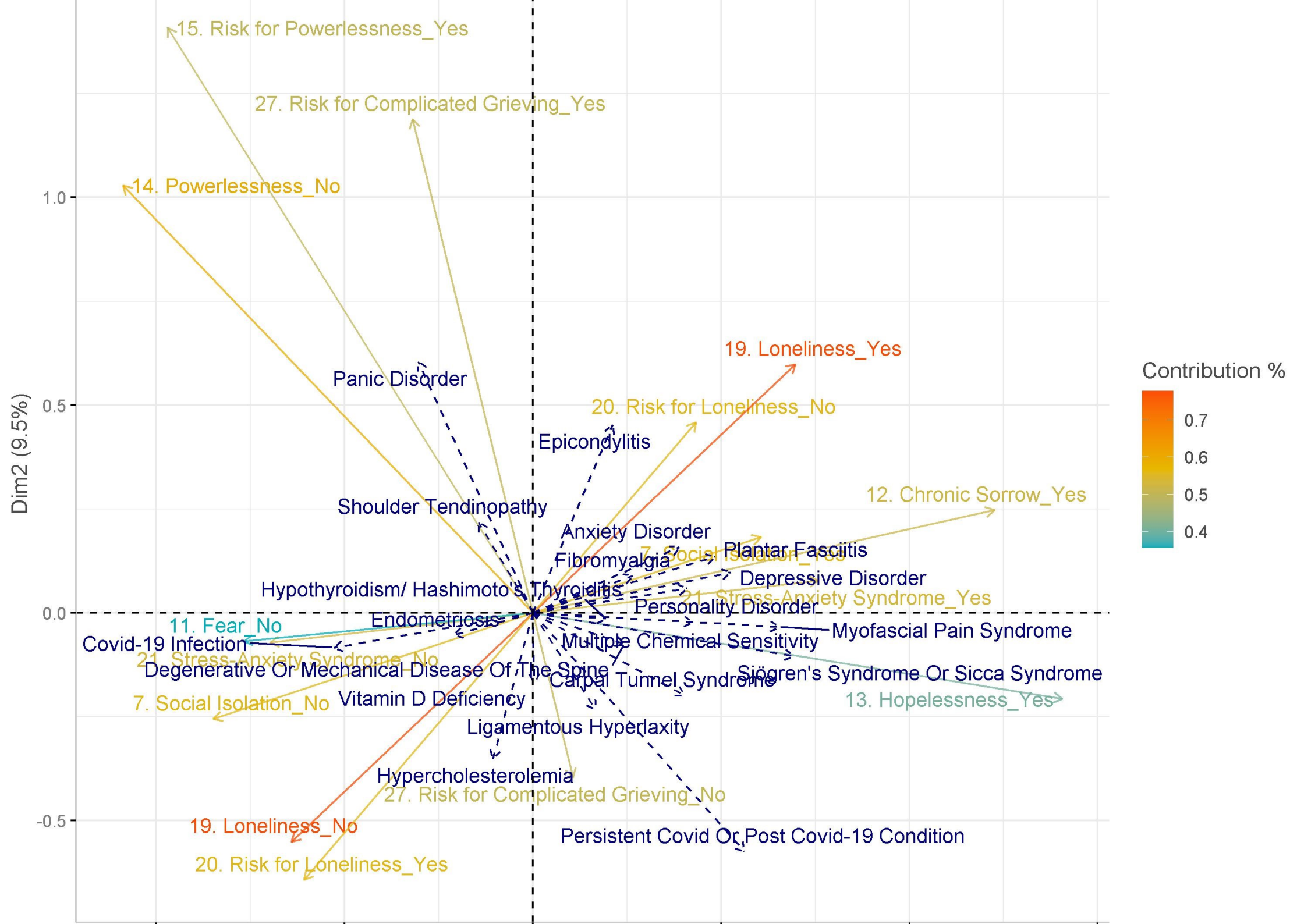

Supplement: Supplementary file 3 — Figure S3. Biplot—MCA. Relationship between psychosocial diagnostic labels and self‐reported current or past health issues. [file NOP2-12-e70212-s002.pdf]
